# Expression of group-II phospholipase A2 in malignant and non-malignant human gastric mucosa.

**DOI:** 10.1038/bjc.1993.294

**Published:** 1993-07

**Authors:** K. Murata, H. Egami, H. Kiyohara, S. Oshima, T. Kurizaki, M. Ogawa

**Affiliations:** Department of Surgery II, Kumamoto University Medical School, Japan.

## Abstract

**Images:**


Br. J. Cancer (1993), 68, 103–111
© Macmillan Press Ltd., 1993

### Expression of group-II phospholipase $A_2$ in malignant and non-malignant human gastric mucosa

K. Murata, H. Egami, H. Kiyohara, S. Oshima, T. Kurizaki & M. Ogawa

*Department of Surgery II, Kumamoto University Medical School, Honjo 1-1-1, Kumamoto, Kumamoto 860, Japan.*

**Summary** The expression of Group-II phospholipase $A_2$ (M-$PLA_2$) was analysed immunohistochemically in malignant, non-malignant (including atrophic, hyperplastic, pseudopyloric metaplastic and intestinal metaplastic) and normal human gastric mucosae. M-$PLA_2$ was consistently detected in the stem cell lineage, pseudopyloric metaplasia and the generative cells of hyperplastic foveolar epithelium and intestinal metaplasia (IM). In IM, the appearance of M-$PLA_2$ was found to be closely related to the degree of development of the brush borders on columnar cells and was especially prominent at dense brush borders. Paneth cells of IM, particularly their secretory products, were strongly immunoreactive for M-$PLA_2$. In gastric cancer, the expression of M-$PLA_2$ was detected exclusively in cancer cells with a low grade of differentiation, and seemed to be intensified in the invading zone of the tumour. These observations suggest that the expression of M-$PLA_2$ is associated with the proliferative kinetics and regeneration of human gastric mucosa, and may indicate a physiological relationship between its expression and metaplasia of small intestinal type. Moreover, the appearance of M-$PLA_2$ may be related to the invasive ability of gastric cancer.

Phospholipase $A_2$ ($PLA_2$) catalyses the specific hydrolysis of a fatty acyl ester bond at the sn-2 position of glycerophospholipids. In addition, a calcium-dependent $PLA_2$ is thought to be one of the most important enzymes regulating the release of arachidonic acid from membrane phospholipids (Lands, 1968; Van den Bosh, 1980). Until recently, two genetically distinct isoenzymes, exocrine $PLA_2$ (secreted in pancreatic juice) and intracellular $PLA_2$ (contained in the cytosol and membrane-associated fraction), have been recognised in humans (Vadas & Pruzanski, 1986; Nakaguchi *et al.*, 1986). In the membrane fraction of human spleen cells the existence of a $PLA_2$, which does not react with anti-human P-$PLA_2$ antibody, has been demonstrated (Nakaguchi *et al.*, 1986). Further purification and subsequent sequencing of this enzyme revealed that it belongs to the Group-II $PLA_2$ (Kanda *et al.*, 1989). Group-II $PLA_2$ has been thought to play an important regulatory role in several metabolic pathways (Nakano *et al.*, 1990*a*; Nakano *et al.*, 1990*b*). And recently, the distribution of this Group-II-$PLA_2$ in human organs has been published (Kiyohara *et al.*, 1992).

The product of this enzyme's action, free arachidonate (which serves as a substrate for the cyclo-oxygenase route and lipoxygenase route) (Van den Bosh, 1980) is the first and rate-limiting precursor in the biosynthes of prostaglandins (PG), leukotriene and HETE (Lands, 1979; Van den Bosh, 1980). Among these products, PG is abundant in gastrointestinal mucosa (Robert *et al.*, 1979), and especially $PGE_2$, has potent cytoprotective effects, e.g. inhibition of gastric secretion, prevention of ulcer formation, and acceleration of the healing of mucosal damage (Robert *et al.*, 1979; Wilson *et al.*, 1971; Robert *et al.*, 1976). Nevertheless, the biological significance and function of $PLA_2$ in the human stomach is still unknown. In the rat gastric mucosa, $PLA_2$ which is structurally identical to the rat pancreatic type $PLA_2$ (P-$PLA_2$, Group-I $PLA_2$) (Tojo *et al.*, 1988; Okamoto *et al.*, 1985) was immunocytochemically detected in chief cells (Tatsumi *et al.*, 1990). Knowledge of the distribution of this enzyme in human gastric mucosa might provide useful insight into the function of $PLA_2$ in gastric mucosa.

In this study, the immunohistochemical expression of M-$PLA_2$ was examined by using a monoclonal antibody (MoAb) against the recently described human splenic Group-II $PLA_2$ (M-$PLA_2$) in a variety of human gastric mucosae, such as normal, atrophic, hyperplastic, pseudopyloric metaplastic and intestinal metaplastic mucosae, as well as cancerous lesions. Moreover, the possible biological role of M-$PLA_2$ is discussed, especially concerning the relationship between its expression and the cell- and tissue-kinetics of gastric mucosa.

Correspondence: M. Ogawa.
Received 19 August 1992; and in revised form 15 January 1993.

### Materials and methods

### *Tissues*

Primary gastric cancers were obtained from 45 surgical specimens of gastrectomised patients. These patients consisted of 27 males and 18 females. Their ages ranged from 32 to 85 years old (average age 66.2 years). Tissues were fixed in 10% buffered formaldehyde for 4 days and embedded in paraffin. One of the serial sections from each tissue was stained with hematoxylin and eosin (H&E) for routine histologic examination, and the others were treated for the demonstration of M-$PLA_2$ as described below. In the 45 specimens examined, normal foveolar epithelia of the cardia, fundus, and antrum were found in two, nine and two cases, respectively. Normal glands of cardia, fundus and antrum were also found in two, six and two cases, respectively. Moreover, normal duodenal mucosae in four cases were also studied.

### *Classification of intestinal metaplastic mucosa (IM)*

Intestinal metaplastic changes in gastric mucosa near and/or adjacent to cancerous lesions were found in 32 cases. Since IM changes were often variable even within a single specimen, the intestinal metaplastic ducts were classified into three groups, according to the degree of development of the brush border on columnar cells (Morson, 1955; Murata *et al.*, 1992). Thus, IMs containing densely, sparsely or undeveloped brush borders were observed in 14, 10 and 20 sites, respectively, in 32 cases.

### *Classification of gastric cancer*

Gastric cancers were morphologically classified into six types according to the General Rules for the Gastric Cancer Study of the Japanese Research Society for Gastric Cancer (Japanese Research Society for Gastric Cancer, 1985); papillary adenocarcinoma (pap), tubular adenocarcinomas of the well ($tub_1$) or moderately ($tub_2$) differentiated type, poorly differentiated adenocarcinoma (por), mucinous adenocarcinoma (muc), and signet-ring cell carcinoma (sig). The 45 gastric cancer tissues were classified a seven pap, eight $tub_1$, ten $tub_2$, 11 por, seven sig, and three muc types.

*Monoclonal antibody (MoAb)*

The MoAb anti-M-$PLA_2$ (IgG) employed was raised in mice immunised against M-$PLA_2$ purified from the membrane-fraction of human spleens (Matsuda *et al.*, 1991).

*Avidin-biotin-peroxidase complex method*

ABC kits (Vector Laboratories, Inc. USA) for mouse IgG were used. Formalin-fixed and paraffin-embedded tissue sections were deparaffinised with xylene and rehydrated with a series of ethanol solutions. Tissue sections were incubated in normal serum for 30 min following the block of endogenous peroxidase activity with 1% $H_2O_2$ in methanol for 20 min, incubated overnight at 4°C with optimally diluted primary MoAb, and subsequently incubated with biotinylated anti-mouse IgG and avidin-biotin peroxidase complex for 30 min at room temperature. They were washed in 0.01 M phosphate-buffered saline (PBS, pH 7.2) between each incubation step. The peroxidase reaction was applied using 0.01% 3,3′-diaminobenzidine tetrahydrochloride (Sigma: St Louis, MO, USA) in 0.05 M Tris-HCl buffer (pH 7.6). Sections were counterstained with Mayer's hematoxylin. For the negative control, the following procedures were employed: (1) sections were processed without the primary antibody, and (2) mouse IgG (M9144, Sigma: St Louis, MO, USA) was used instead of the primary antibody.

### Results

The distribution of the M-$PLA_2$-immunoreactive cells in human gastric and duodenal mucosa is summarised in Table I.

*Normal gastric and duodenal mucosa*

M-$PLA_2$ was not expressed in the normal foveolar epithelia of two cardiac, nine fundic, and two antral mucosae. Similarly, M-$PLA_2$ was not detected in two cardiac, six fundic, and two antral proper glands.

In four normal duodenal mucosae, the foveolar epithelia in all four specimens and Brunner's glands in two specimens expressed this antigen (Figure 1). Furthermore, almost all Paneth cells at the bottom of these crypts expressed M-$PLA_2$

**Table I** Distribution of M-$PLA_2$ immunoreactive cells in non-malignant gastric and duodenal mucosa

| | *No. of positive staining/No of sites* |
|---|---|
| 1. Normal gastric mucosa | |
| Foveolar epithelia | |
| cardiac | 0/2 |
| fundic | 0/9 |
| antral | 0/2 |
| Gland | |
| cardiac | 0/2 |
| fundic | 0/6 |
| antral | 0/2 |
| 2. Atrophic mucosa | |
| Foveolar epithelia | |
| cardiac | 0/0 |
| fundic | 0/3 |
| antral | 0/1 |
| Gland | |
| cardiac | 0/0 |
| fundic | 0/16 |
| antral | 0/2 |
| 3. Hyperplastic mucosa | |
| Foveolar epithelia | |
| cardiac | 0/0 |
| fundic | 1/2 (50.0%) |
| antral | 0/1 |
| Gland | |
| cardiac | 0/0 |
| fundic | 0/3 |
| antral | 0/1 |
| 4. Pseudopyloric metaplasia | 22/26 (84.6%) |
| 5. Generative zone | 16/20 (80.0%) |
| 6. Intestinal metaplasia | 14/44 (45.5%) |
| 7. Normal duodenal mucosa | |
| Foveolar epithelia | 4/4 (100%) |
| Brunner's gland | 2/2 (100%) |

**Figure 1** Immunohistochemical staining for M-$PLA_2$ in duodenal mucosa in Golgi pattern. Bar = 100 μm.

in all four specimens. The expression of M-PLA$_2$ in foveolar epithelia and Brunner's glands were found in Golgi and cytoplasmic pattern, respectively. In Paneth cells, positive staining in a fine granular pattern was seen among the eosinophilic granules but not in the cytoplasm. In a few cases, mucous neck cells, which are localised to an isthmus of gastric mucosa, were found to faintly express this antigen in cytoplasmic granular pattern (Figure 2).

### *Atrophic mucosa and pseudopyloric metaplasia*

Three fundic and one antral epithelia exhibiting atrophic changes were found not to express M-PLA$_2$. Similarly, the atrophic glands of 16 fundic and two antral mucosae were also negative.

Pseudopyloric metaplasia, which appears as one of the regenerative forms following mucosal damage from gastritis or ulceration, was observed in 26 cases, and in 22 (84.6%) cases, M-PLA$_2$ was expressed in Golgi pattern (Figure 3). Furthermore, M-PLA$_2$ appeared in Golgi pattern in 12 out of 14 (85.7%) cases with cystic degeneration of the pseudopyloric glands.

### *Hyperplastic mucosa*

Hyperplasia of the foveolar epithelia in the fundus and antrum was observed in two and one case, respectively, and the expression of M-PLA$_2$ was seen in one fundic epithelium in the Golgi region. At the isthmus of this positive mucosa, which is postulated to be the generative zone of this hyperplastic epithelium, a strong immunoreactivity in Golgi or cytoplasmic granular pattern was observed (Figure 4a). As for hyperplasia of the proper glands, M-PLA$_2$ was not expressed in three fundic nor one antral mucosae.

**Figure 3** Immunohistochemical staining for M-PLA$_2$ in a patient with pseudopyloric metaplasia. The stain is distributed in a Golgi pattern. This pattern was also seen in epithelia exhibiting an absorptive-type metaplasia as well as in cystic degeneration of the pseudopyloric glands. The 'ABC' method described in the text was used. Bar = 100 μm.

**Figure 2** Immunohistochemical staining for M-PLA$_2$ in the mucous neck cells at the isthmus of gastric mucosa in Golgi pattern. Bar = 100 μm.

### *Generative zone*

When regenerative transformation occurs following mucosal damage caused by gastritis, erosion, and ulceration, elongation of the generative zone, which consists of generative cells, is known to occur (Wright *et al.*, 1990). In this study, an apparent generative zone was found in 20 cases, and M-PLA$_2$ was demonstrated in 16 (80.0%) cases in Golgi or cytoplasmic granular pattern (Figure 4b). M-PLA$_2$ was often detected in Golgi or cytoplasmic granular pattern at the bottom of the negative intestinal metaplastic ducts as well as above the generative zone (Figure 4c).

### *Intestinal metaplastic ducts and Paneth cells*

In IM near and/or adjacent to cancerous lesions, the frequency of the expression of M-PLA$_2$ in crypts with densely, sparsely and undeveloped brush borders was 14/14 (100%), 6/10 (60.0%) and 0/20 (0%) sites, respectively. All the positive staining of the columnar cells occurred in Golgi pattern. Moreover, at dense brush borders, the manifest appearance of M-PLA$_2$ was often demonstrated (Figure 5a). On the other hand, all of the goblet cells were negative.

Twenty-two cases of those with intestinal metaplastic ducts had Paneth cells at and/or near the bottom of their crypts. Twenty (87.0%) cases exhibited positive staining, and M-PLA$_2$ expression was observed not in the cytoplasm but among the many secretory granules, as well as in the duodenal mucosa (Figure 5b).

### *Cancerous tissues*

As shown in Table II, the frequency of M-PLA$_2$ expression was inversely proportional to the degree of cell-differentiation. The expression of this antigen was negative in seven papillary adenocarcinomas. Positive staining was found in one out of eight (12.5%) well differentiated tubular adenocarcinomas (tub$_1$) (Figure 6a).

In contrast, M-PLA$_2$ was frequently expressed in the more poorly differentiated cancer cells, such as the poorly

**Figure 4** Immunohistochemical staining for M-$PLA_2$ in the generative cells of gastric mucosa in Golgi or cytoplastic granular pattern. Positive staining was observed **a**, at the isthmus of the positive hyperplastic foveolar epithelium. Bar = 100 μm; **b**, in the elongated zone of generative cells. Bar = 50 μm; **c**, in the bottom of negative intestinal metaplastic ducts without dense brush border. Bar = 100 μm.

**Figure 5** **a**, Immunohistochemical staining for M-PLA$_2$ in a patient with IM and a dense brush border. There is prominent staining of the brush border. There is not detectable staining of the goblet cells. Bar = 100 μm. **b**, Immunohistochemical staining for M-PLA$_2$ in a patient with IM and many Paneth cells. Heavy staining is seen among the many secretory granules of the Paneth cells. Bar = 50 μm.

differentiated adenocarcinomas (por) and the moderately differentiated tubular adenocarcinomas (tub$_2$). Positive staining in por and tub$_2$ was found in eight out of 11 (72.7%) and four out of ten (40.0%) cases, respectively, and their staining pattern was cytoplasmic granular (Figure 6b). The expression of M-PLA$_2$ seemed to be more pronounced in the invasive areas of the tumour (Figure 6c). On the other hand, cancer cells capable of producing mucin, such as signet-ring cell carcinoma (sig) and mucinous adenocarcinoma (muc), generally failed to stain with the monoclonal antibody, although a few sig cells which were interspersed among por faintly expressed M-PLA$_2$.

### Discussion

In the present study, the expression of M-$PLA_2$ was analysed immunohistochemically in malignant and non-malignant mucosae (including atrophic, hyperplastic, pseudopyloric metaplastic and intestinal metaplastic), as well as normal mucosa of the human stomach.

In normal gastric mucosa, M-$PLA_2$ was expressed in neither foveolar epithelia nor proper glands, except for mucous neck cells which are thought to be generative cells (stem cells) in gastric mucosa (MacDonald *et al.*, 1964). In contrast, the elongated zone of stem cells, which appears following a breach in the gastrointestinal mucosa (Wright *et al.*, 1990), strongly expressed M-$PLA_2$ in the same pattern.

**Table II** M-$PLA_2$-expression and histopathological types of gastric cancer

| | *pap* | $tub_1$ | $tub_2$ | *por* | *sig* | *muc* |
|---|---|---|---|---|---|---|
| | 0/7[a] | 1/8[a] | 4/10 | 8/11[a] | 0/7 | 0/3 |
| % | 0 | 12.5 | 40.0 | 72.7 | 0 | 0 |

Exact probability test: [a]$P < 0.01$.

c

**Figure 6** **a**, Immunohistochemical staining for M-PLA$_2$ in a patient with a well differentiated gastric cancer. No significant staining is observed except for some positive staining in a residual cystic pseudopyloric gland. Bar = 100 µm. **b**, Immunohistochemical staining for M-PLA$_2$ in a patient with an undifferentiated gastric cancer. Significant staining is observed in cytoplasmic granular pattern. Bar = 100 µm. **c**, Immunohistochemical staining for M-PLA$_2$ in a patient with an invasive, undifferentiated gastric cancer. Note the intensive staining observed in the invading zone of the tumour. Bar = 100 µm.

Similarly, the isthmus in hyperplastic foveolar epithelia, which is postulated to be its generative zone, exhibited significant M-PLA$_2$ expression. In intestinal metaplasia, M-PLA$_2$ was expressed in cells at the bottom of the glands, differing from the Paneth cells found in M-PLA$_2$-negative metaplastic ducts. Since the generative cells in IM have been reported to be localised at the bottom of the ducts, these positive cells were thought to be identical with generative cells of IM (Hattori & Fujita, 1979; Poulsen *et al.*, 1986). Furthermore, pseudopyloric glands, which signify gastric regeneration (Hashimoto *et al.*, 1983), frequently expressed this enzyme. These results suggest an association of the presence of M-PLA$_2$ with cell proliferative kinetics and regeneration of gastric mucosa. Moreover, it is tempting to postulate that the increased expression of PLA$_2$, stemming from a mucosal accident or age-related changes, results in the increased biosynthesis of prostaglandins which protects the gastric mucosa and accelerates the healing of the mucosa damage (Robert *et al.*, 1979; Wilson *et al.*, 1971; Robert *et al.*, 1976).

M-PLA$_2$ was frequently expressed in IM. However, IM is not a single entity but rather variable by a number of criteria including morphologic (Morson, 1955), enzymatic (Shimada *et al.*, 1987), and mucin histochemical studies (Segura & Montero, 1983). For example, small and large intestinal types (or complete and incomplete) have been described. In this study, IM was morphologically classified into three groups, according to the degree of development of the brush border. We found a positive association between the expression of M-PLA$_2$ and the extent of development of the brush border, and dense brush borders often had substantial positive staining. In a separate study, we found that M-PLA$_2$ was distributed in the normal human digestive tract mainly in small intestine in a pattern similar to that seen in IM. It was not detected in the large intestine. This suggests that the cells in the metaplastic ducts expression M-PLA$_2$ are physiologically related to those in the normal small intestine which express M-PLA$_2$. Therefore, the results in this study may indicate the association with the appearance of M-PLA$_2$ and the transformation into intestinal type of gastric mucosa. And the presence of M-PLA$_2$ positive cells in IM might enable one to classify it as a 'small intestinal' or 'complete' type.

The expression of M-PLA$_2$ in a granular pattern was observed among many secretory vesicles in almost all Paneth cells, behaving as secretory cells. This appearance in Paneth cells is consistent with a previous report which demonstrated that Paneth cells secrete some digestive enzymes (Senagas-Balas *et al.*, 1984). Since the Paneth cell is postulated to secrete substances affecting the growth and differentiation of intestinal epithelia (Poulsen *et al.*, 1986), the expression of M-PLA$_2$ might be related to cell proliferation not only in the normal intestine but also in IM.

In addition, the secretory granules in the Paneth cells of germ-free mammalian small intestine have been reported to change subsequent to the administration of bacteria (Satoh, 1988). This hints that Paneth cells play some role in the regulation of the bacterial milieu in the small intestine. It has been previously demonstrated that there is no cross-immunoreactivity between pancreatic and intestinal phospholipase in rat Paneth cells (Erlandsen & Case, 1972). Therefore, these results suggest that the cell membrane of bacteria could be denatured by M-PLA$_2$ expressed in Paneth cells and the bacterial milieu might be regulated in the small intestine and also in the complete type of IM. Moreover, the hydrolysis catalysed by M-PLA$_2$ may be a necessary step for the phagocytosis of bacteria (Matsukura *et al.*, 1979).

In a separate study, we found that M-PLA$_2$ was expressed in the foetal but not the adult gastric mucosa (Kiyohara *et al.*, 1992). Therefore, the abundant expression of M-PLA$_2$ in undifferentiated cancer cells supports the hypothesis that the presence of this enzyme may be related to cell- and/or tissue-differentiation of gastric mucosa. The expression of M-PLA$_2$ in cancer was inversely proportional to the degree of the carcinoma's cell-differentiation. Particularly intense staining was seen in invasive gastric cancer as well as poorly differentiated adenocarcinoma (por). The staining was most pronounced at the invading zone near the centre of the cancerous tissue. These results suggest that poorly differentiated cancer cells may require the hydrolysis catalysed by M-PLA$_2$ to infiltrate into uninvolved and adjacent tissues.

IM has been recognised as a precursor of gastric cancer by a number of morphologic (Morson, 1955), enzymatic (Shimada *et al.*, 1987), mucin histochemical (Segura & Montero, 1983), and experimental studies (Matsukura, 1979). This is especially true of the apparent relationship between differentiated cancer, such as pap and $tub_1$, and the large intestinal (incomplete) type of IM (Murata *et al.*, 1992; Shimada *et al.*, 1987). In contrast, there have been few reports concerning the precursors of undifferentiated gastric cancer. In the present study, M-$PLA_2$ was absent in both most of differentiated cancers and the incomplete type of IM lacking Paneth cells and a brush border. However, it was strongly positive in both undifferentiated cancer and in the generative cells of the stomach. Undifferentiated por cancer (but not sig) is thought to originate from the undifferentiated cell zone of the non-metaplastic mucosa (Hattori, 1985). Therefore, these results indicate that the generative cells may be the precursor cells of undifferentiated gastric cancer.

Since Levine *et al.* (1977) first reported the release of $PGE_2$ and $PGF_{2a}$ from renal cells stimulated by epidermal growth factor (EGF), it has been demonstrated that various cytokines, e.g. EGF (Nolan *et al.*, 1988), tumour necrosis factor (Hori *et al.*, 1989) and interleukin-1 (Dayer *et al.*, 1990) stimulate $PLA_2$-activity and induce the synthesis of certain prostaglandins which regulate cell mitogenesis. Therefore, the growth of M-$PLA_2$-positive cancer cells, especially those which are undifferentiated might be regulated by prostaglandins via the activation of M-$PLA_2$. Additionally, since some kinds of fibroblasts have been reported to proliferate in response to prostaglandins (Nolan *et al.*, 1988; Handler *et al.*, 1990), prostaglandins induced by M-$PLA_2$-activation could accelerate the growth of interstitial tissue, resulting in the scirrhous changes usually observed in advanced undifferentiated cancer. Furthermore, M-$PLA_2$ itself could be directly related to growth and differentiation in the human gastrointestinal tract, since P-$PLA_2$ was recently reported to be a growth factor (Arita *et al.*, 1991).

### References

ARITA, H., HANASAKI, K., NAKANO, T., OKA, S., TERAOKA, H. & MATSUMOTO, K. (1991). Novel proliferative effect of phospholipase $A_2$ in Swiss 3T3 cells via specific binding site. *J. Biol. Chem.*, **266**, 19139–19141.

DAYER, J.M., BEUTLER, B. & CERAMI, A. (1985). Cachectin/tumor necrosis factor stimulates collagenase and prostaglandin $E_2$ production by human synovial cells and dermal fibroblasts. *J. Exp. Med.*, **162**, 2163–2168.

ERLANDSEN, S.L. & CASE, D.G. (1972). Paneth cell function: phagocytosis and intracellular digestion of intestinal microorganism. I. Hexamita muris. *J. Ultrastruct. Res.*, **41**, 296–318.

HANDLER, J.A., DANILOWICZ, R.M. & ELING, T.E. (1990). Mitogenic signaling by epidermal growth factor (EGF), but not platelet-derived growth factor, requires arachidonic acid metabolism in BALB/c 3T3 cells. *J. Biol. Chem.*, **265**, 3669–3673.

HASHIMOTO, M., TOKUNAGA, A., NISHI, K., WADA, M., MASUMORI, K., KUNAGAE, Y., NUMAJIRI, H., MATSUKURA, N., YOSHIYASU, M., TANAKA, N., SHIROTA, A. & ASANO, G. (1983). [$^3$H]Thymidine autoradiographic and alkaline phosphatase histochemical studies of intestinal metaplasia of the human stomach. *Histochem. J.*, **15**, 953–959.

HATTORI, T. & FUJITA, S. (1979). Tritiated thymidine autoradiographic study on histogenesis and spreading of intestinal metaplasia in human stomach. *Path. Res. Pract.*, **164**, 224–237.

HATTORI, T. (1985). Histological and autoradiographic study on development of group III lesion (dysplasia grade III) in the stomach. *Path. Res. Pract.*, **180**, 36–44.

HORI, T., KISHIYAMA, S., HAYAKAWA, M., SHIBAMOTO, S., TSUJIMOTO, M., OKU, N. & ITO, F. (1989). Possible role of prostaglandins as negative regulators in growth stimulation by tumor necrosis factor and epidermal growth factor in human fibroblasts. *J. Cell. Physiol.*, **141**, 275–280.

JAPANESE RESEARCH SOCIETY FOR GASTRIC CANCER: GENERAL RULES FOR THE GASTRIC CANCER STUDY (1985). Kanehara, Tokyo (11th ed.).

KANDA, A., ONO, T., YOSHIDA, N., TOJO, H. & OKAMOTO, M. (1989). The primary structure of a membrane-associated phospholipase $A_2$ from human spleen. *Biochem. Biophys. Res. Commun.*, **163**, 42–48.

KIYOHARA, H., EGAMI, H., SHIBATA, Y., MURATA, K., OSHIMA, S. & OGAWA, M. (1992). Light microscopic immunohistochemical analysis of the distribution of group II phospholipase $A_2$ in human digestive organs. *J. Histochem. Cytochem.*, **40**, 1659–1664.

LANDS, W.E.M. (1968). The biosynthesis and metabolism of prostaglandins. *Biochim. Biophys. Acta.*, **164**, 426–429.

LANDS, W.E.M. (1979). The biosynthesis and metabolism of prostaglandins. *Annu. Rev. Physiol.*, **41**, 633–652.

LEVINE, L. & HASSID, A. (1977). Epidermal growth factor stimulates prostaglandin biosynthesis by canine kidney (MDCK) cells. *Biochem. Biophys. Res. Commun.*, **76**, 1181–1187.

MACDONALD, W.C., TRIER, J.S. & EVERETT, N.B. (1964). Cell proliferation and migration in the stomach, duodenum and rectum of man: radioautographic studies. *Gastroenterology*, **46**, 721–729.

MATSUDA, Y., OGAWA, M., SAKAMOTO, K., YAMASHITA, S., KANDA, A., KOHNO, M., YOSHIDA, N., NISHIJIMA, J., MURATA, A. & MORI, T. (1991). Development of a radioimmunoassay for human group II phospholipase $A_2$ and demonstration of postoperative elevation. *Enzyme*, **45**, 200–208.

MATSUKURA, N., KAWACHI, T. & SUGIMURA, T. (1979). Induction of intestinal metaplasia and carcinoma in the grandular stomach of rats by N-alkyl-N′-nitro-soguanidines. *Gann (Jpn. J. Cancer Res.)*, **70**, 181–185.

MORSON, B.C. (1955). Carcinoma arising from areas of intestinal metaplasia in the gastric mucosa. *Br. J. Cancer*, **9**, 377–385.

MURATA, K., EGAMI, H., SHIBATA, Y., SAKAMOTO, K., MISUMI, A. & OGAWA, M. (1992). Expression of blood group-related antigens, ABH, Lewis$^a$, Lewis$^b$, Lewis$^x$, Lewis$^y$, CA19-9, and CSLEX1 in early cancer, intestinal metaplasia, and uninvolved mucosa of the stomach. *Am. J. Clin. Pathol.*, **98**, 67–75.

NAKAGUCHI, K., NISHIJIMA, J., OGAWA, M., MORI, T., TOJO, H., YAMANO, T. & OKAMOTO, M. (1986). Purification and some properties of membrane-associated phospholipase $A_2$ of human spleen. *Enzyme*, **35**, 2–12.

NAKANO, T., OHARA, O., TERAOKA, H. & ARITA, H. (1990*a*). Glucocorticoids suppress Group-II phospholipase $A_2$ production by blocking mRNA synthesis and post-transcriptional expression. *J. Biol. Chem.*, **265**, 12745–12748.

NAKANO, T., OHARA, T., TERAOKA, H. & ARITA, H. (1990*b*). Group II phospholipase $A_2$ mRNA synthesis is stimulated by two distinct mechanisms in rat vascular smooth muscle cells. *FEBS Lett.*, **261**, 171–174.

NOLAN, R.D., DANILOWICZ, R.M. & ELING, T.E. (1988). Role of arachidonic acid metabolism in the mitogenic response of BALB/c 3T3 fibroblasts in epidermal growth factor. *Mol. Pharm.*, **33**, 650–656.

OKAMOTO, M., ONO, T., TOJO, H. & YAMANO, T. (1985). Immunochemical relatedness between secretory phospholipase $A_2$ and intracellular phospholipase $A_2$. *Biochem. Biophys. Res. Commun.*, **2**, 788–794.

POULSEN, S.S., NEXO, E., OLSEN, P.S., HESS, J. & KIRKEGAARD, P. (1986). Immuno-histochemical localization of epidermal growth factor in rat and man. *Histochemistry*, **85**, 389–394.

ROBERT, A., SCHULTZ, J.R., NEZAMIS, J.E. & LANCASTER, C. (1976). Gastric antisecretory and antiulcer properties of $PGE_2$, 15-methyl $PGE_2$ and 16, 16-dimethyl $PGE_2$. Intravenous, oral and intrajejunal administration. *Gastroenterology*, **70**, 359–370.

ROBERT, A., NEZAMIS, J.E., LANCASTER, C. & HANCHAR, A.J. (1979). Cytoprotection by prostaglandins in rats: prevention of gastric necrosis produced by alcohol, HC1, NaOH, hypertonic NaCl, and thermal injury. *Gastroenterology*, **77**, 433–443.

SATOH, Y. (1988). Effect of live and heat-killed bacteria on the secretory activity of Paneth cells in germ-free mice. *Cell. Tissue. Res.*, **251**, 87–93.

SEGURA, D.I. & MONTERO, C. (1983). Histochemical characterization of different types of intestinal metaplasia in gastric mucosa. *Cancer*, **52**, 498–503.

SENEGAS-BALAS, F., BALAS, D., VERGER, R., DE CARO, A., FIGARELLA, C., FERRATO, F., LECHENE, P., BERTRAND, C. & RIBET, A. (1984). Immunohistochemical localization of intestinal phospholipase $A_2$ in rat Paneth cells. *Histochemistry*, **81**, 581–584.

SHIMADA, S., MAENO, M., MISUMI, A. & AKAGI, M. (1987). Antigen reversion of glycogen phosphorylase isoenzyme in carcinoma and proliferative zone of intestinal metaplasia of the human stomach. *Gastroenterology*, **93**, 35–40.

TATSUMI, H., TOJO, H., SENDA, T., ONO, T., FUJITA, H. & OKAMOTO, M. (1990). Immunocytochemical studies on the localization of pancreatic-type phospholipase $A_2$ in rat stomach and pancreas, with special reference to the stomach cells. *Histochemistry*, **94,** 135–140.

THOMPSON, J.F. (1988). Specific receptors for epidermal growth factor in rat intestinal microvillus membranes. *Am. J. Physiol.*, **254,** 429–435.

TOJO, H., ONO, T., KURAMITSU, S., KAGAMIYAMA, H. & OKAMOTO, M. (1988). A phospholipase $A_2$ in the supernatant fraction of rat spleen: its similarity to rat pancreatic phospholipase $A_2$. *J. Biol. Chem.*, **263,** 5724–5731.

VADAS, P. & PRUZANSKI, W. (1986). Biology of disease: role of secretory phospholipase $A_2$ in the pathobiology of disease. *Lab. Invest.*, **55,** 391–404.

VAN DEN BOSCH, H. (1980). Intracellular phospholipase A. *Biochim. Biophys. Acta*, **604,** 191–246.

WILSON, D.E., PHILLIPS, C. & LEVINE, R.A. (1971). Inhibition of gastric secretion in man by prostaglandin $A_1$. *Gastroenterology*, **61,** 201–206.

WRIGHT, N.A., PIKE, C. & ELLIA, G. (1990). Induction of a novel epidermal growth factor-secreting cell lineage by mucosal ulceration in human gastro-intestinal stem cells. *Nature*, **343,** 82–85.


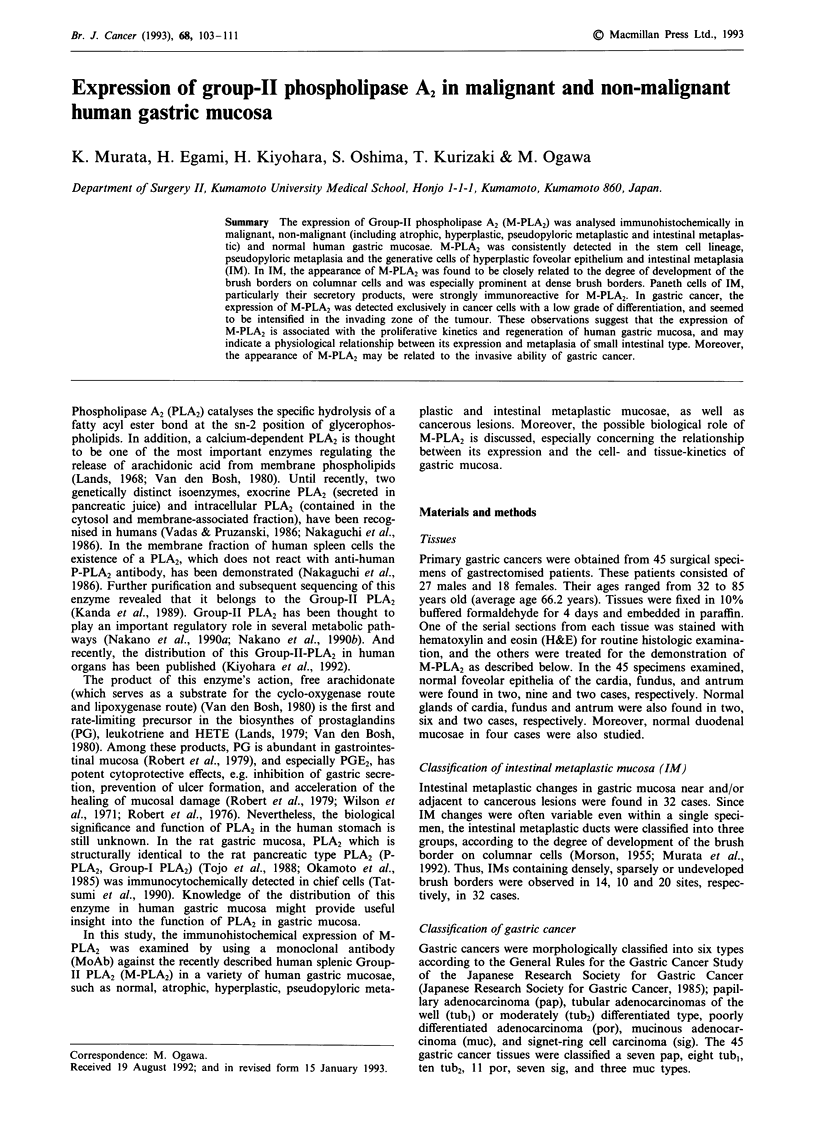

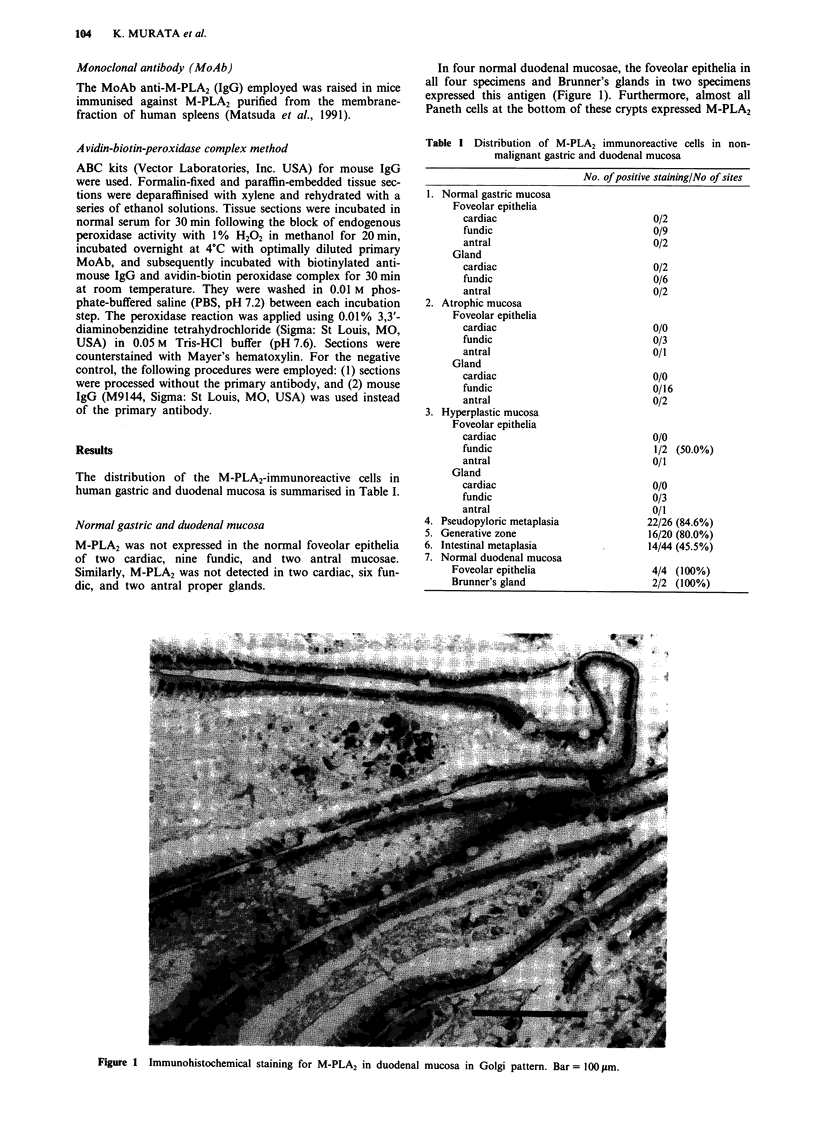

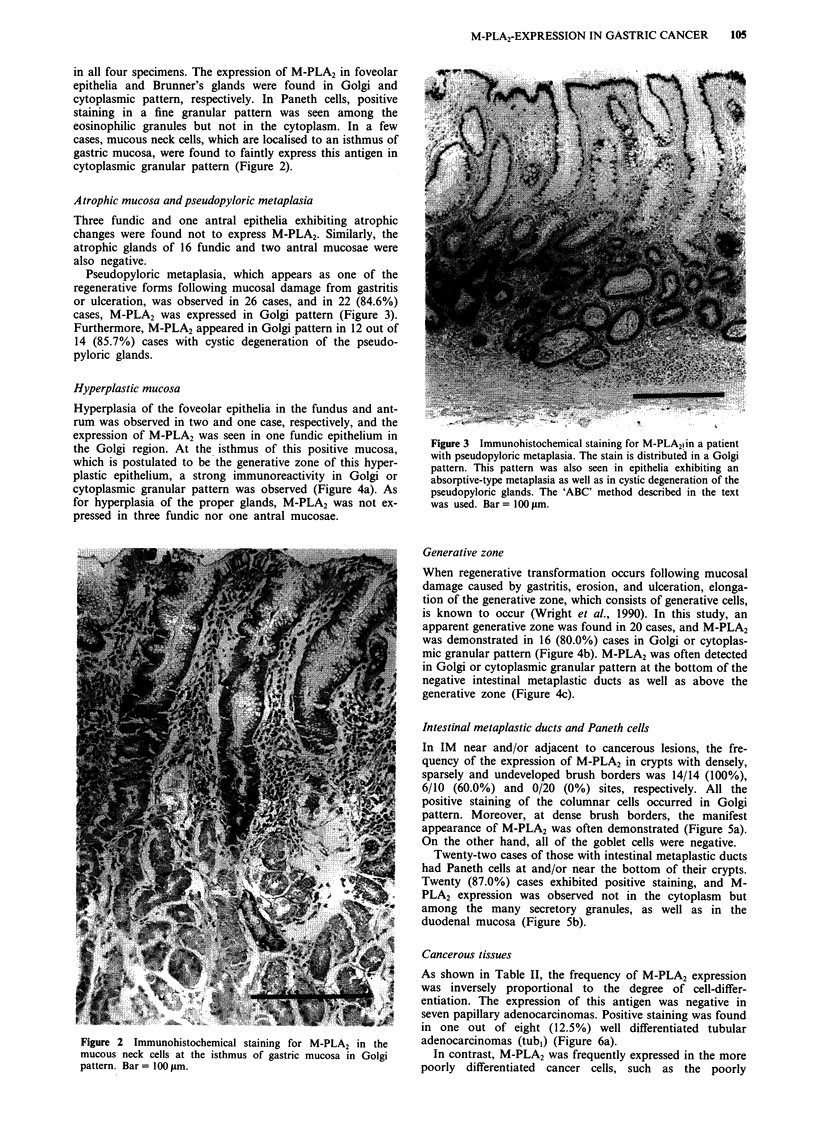

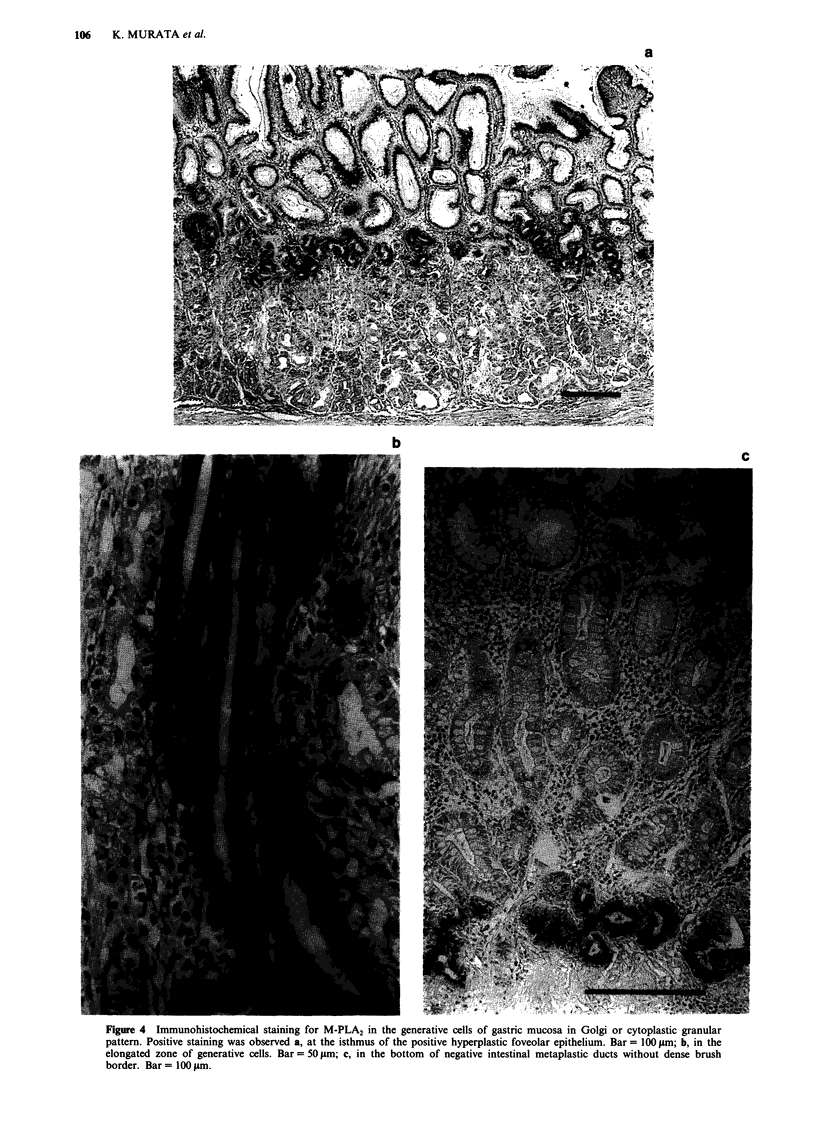

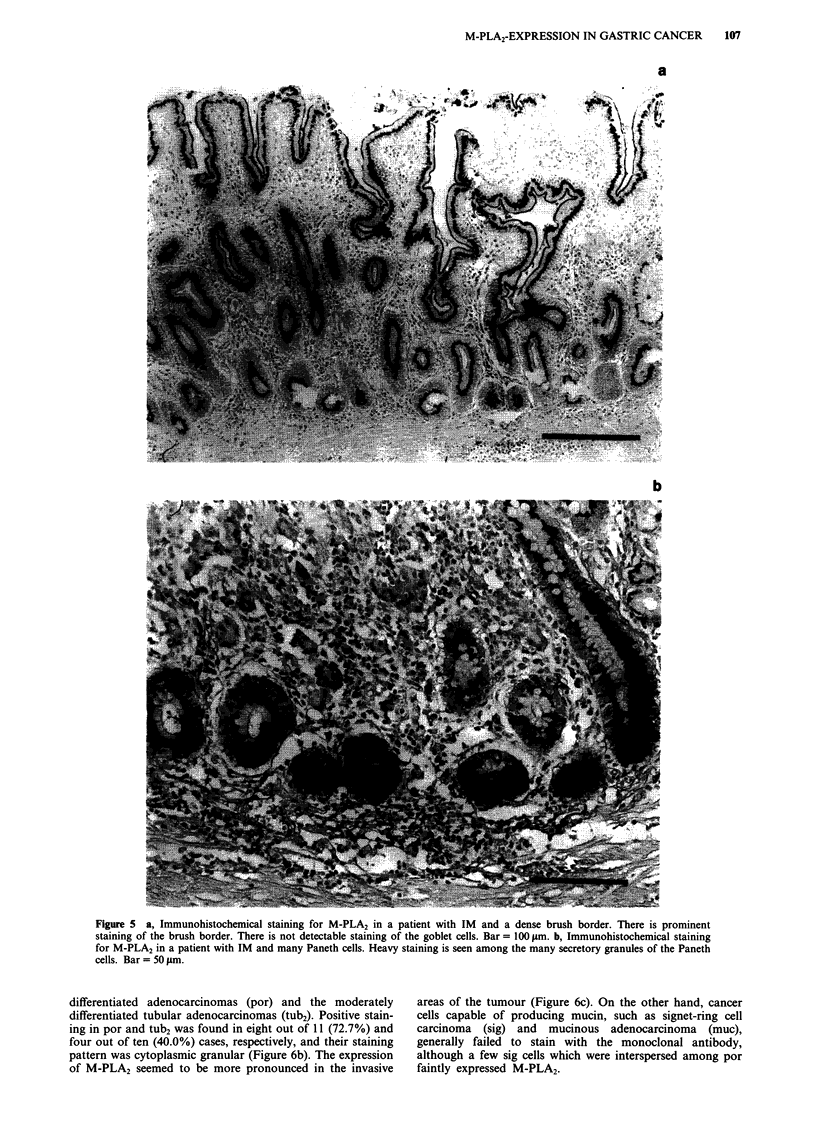

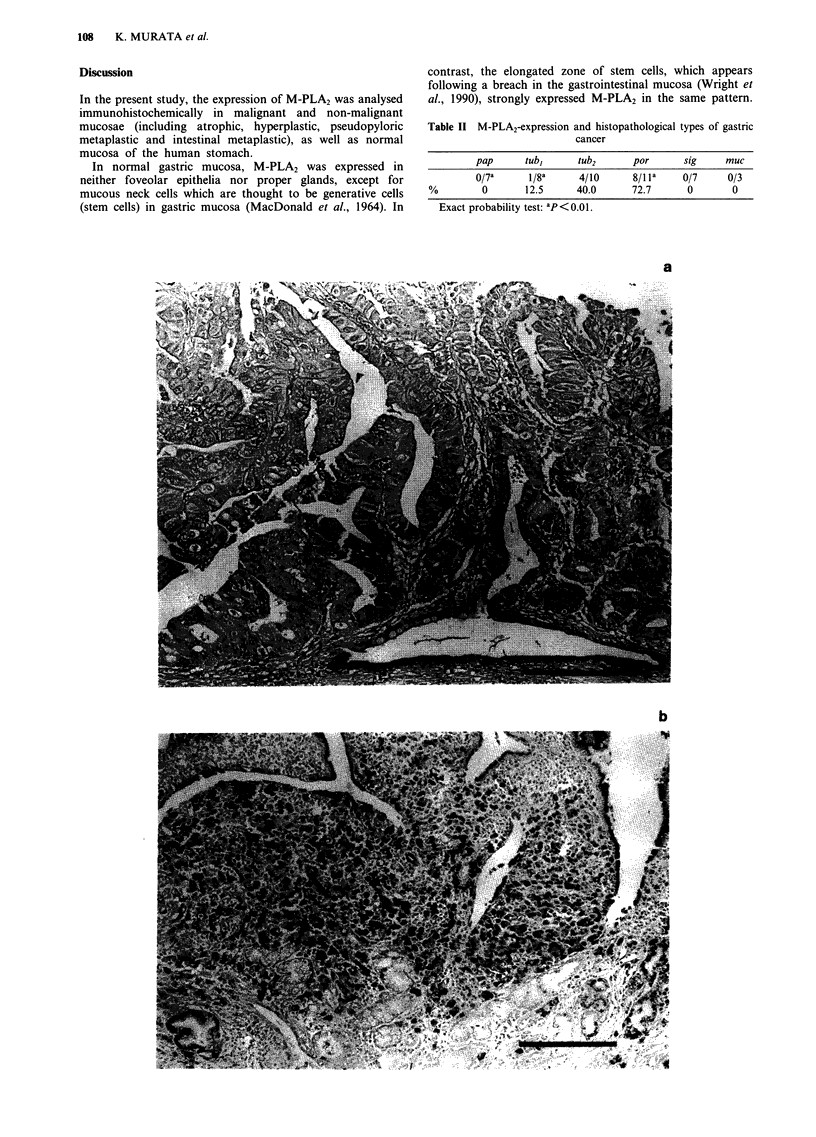

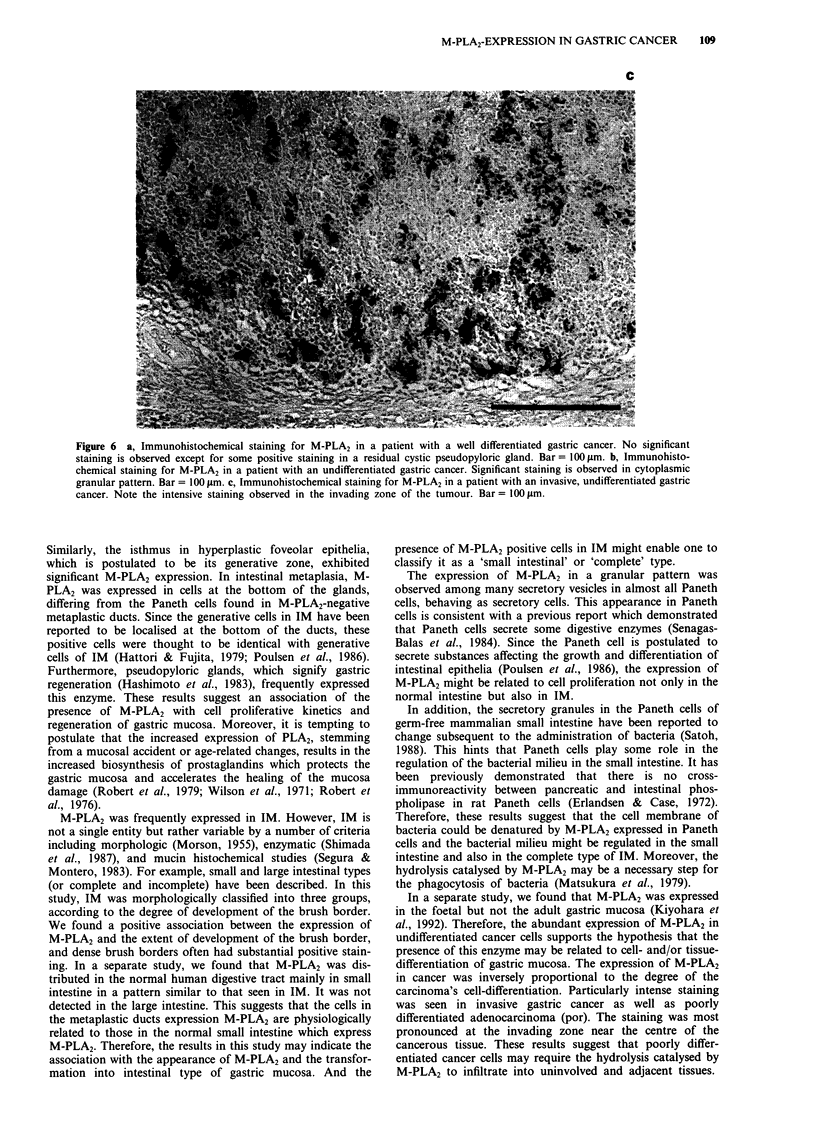

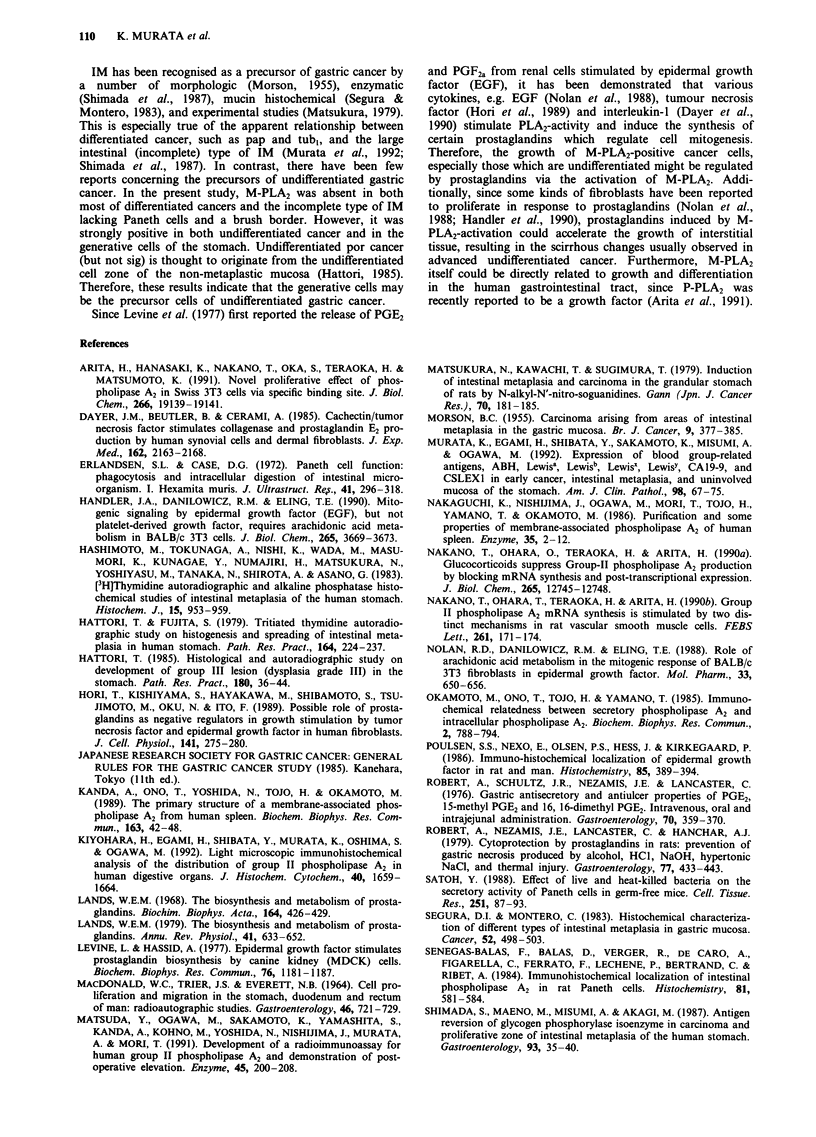

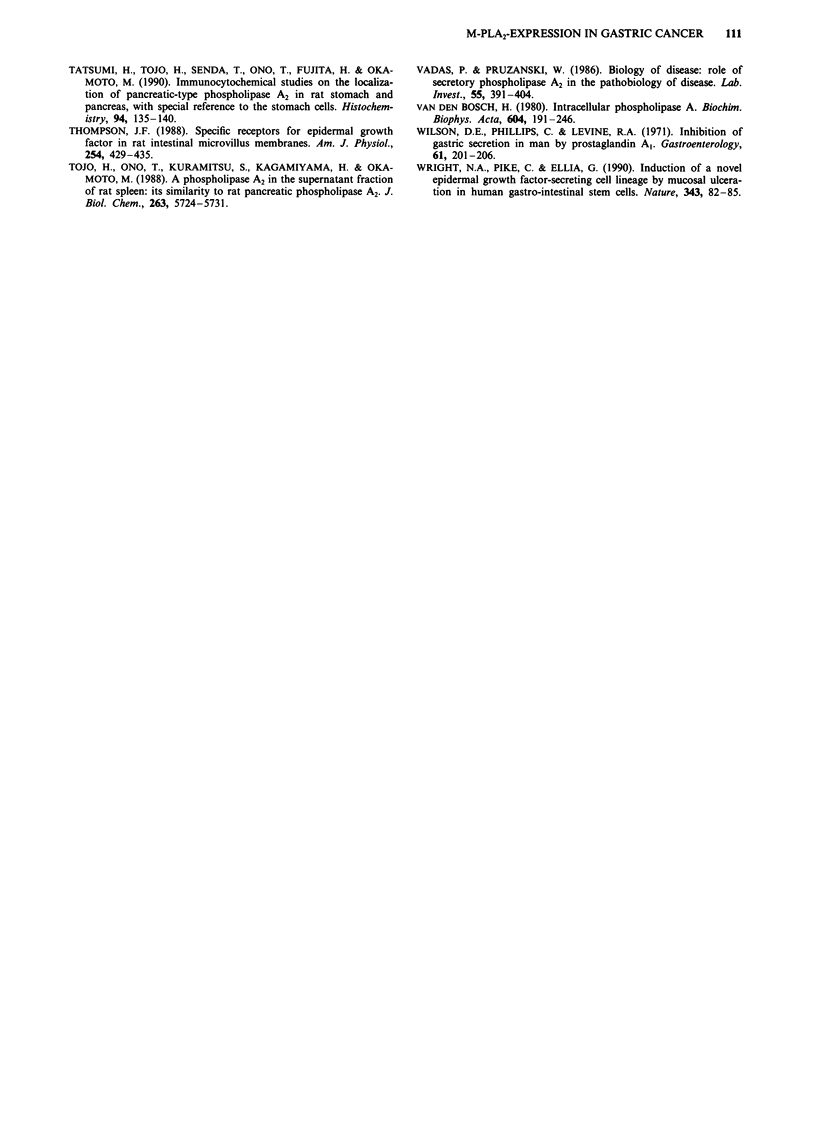


## References

[OCR_00570] Arita H., Hanasaki K., Nakano T., Oka S., Teraoka H., Matsumoto K. (1991). Novel proliferative effect of phospholipase A2 in Swiss 3T3 cells via specific binding site.. J Biol Chem.

[OCR_00576] Dayer J. M., Beutler B., Cerami A. (1985). Cachectin/tumor necrosis factor stimulates collagenase and prostaglandin E2 production by human synovial cells and dermal fibroblasts.. J Exp Med.

[OCR_00582] Erlandsen S. L., Chase D. G. (1972). Paneth cell function: phagocytosis and intracellular digestion of intestinal microorganisms. I. Hexamita muris.. J Ultrastruct Res.

[OCR_00586] Handler J. A., Danilowicz R. M., Eling T. E. (1990). Mitogenic signaling by epidermal growth factor (EGF), but not platelet-derived growth factor, requires arachidonic acid metabolism in BALB/c 3T3 cells. Modulation of EGF-dependent c-myc expression by prostaglandins.. J Biol Chem.

[OCR_00594] Hashimoto M., Tokunaga A., Nishi K., Wada M., Masumori K., Kumagae Y., Numajiri H., Matsukura N., Yoshiyasu M., Tanaka N. (1983). [3H]thymidine autoradiographic and alkaline phosphatase histochemical studies of intestinal metaplasia of the human stomach.. Histochem J.

[OCR_00600] Hattori T., Fujita S. (1979). Tritiated thymidine autoradiographic study on histogenesis and spreading of intestinal metaplasia in human stomach.. Pathol Res Pract.

[OCR_00605] Hattori T. (1985). Histological and autoradiographic study on development of group III lesion (dysplasia grade III) in the stomach.. Pathol Res Pract.

[OCR_00612] Hori T., Kashiyama S., Hayakawa M., Shibamoto S., Tsujimoto M., Oku N., Ito F. (1989). Possible role of prostaglandins as negative regulators in growth stimulation by tumor necrosis factor and epidermal growth factor in human fibroblasts.. J Cell Physiol.

[OCR_00622] Kanda A., Ono T., Yoshida N., Tojo H., Okamoto M. (1989). The primary structure of a membrane-associated phospholipase A2 from human spleen.. Biochem Biophys Res Commun.

[OCR_00628] Kiyohara H., Egami H., Shibata Y., Murata K., Ohshima S., Ogawa M. (1992). Light microscopic immunohistochemical analysis of the distribution of group II phospholipase A2 in human digestive organs.. J Histochem Cytochem.

[OCR_00635] Lands W. E., Samuelsson B. (1968). Phospholipid precursors of prostaglandins.. Biochim Biophys Acta.

[OCR_00639] Lands W. E. (1979). The biosynthesis and metabolism of prostaglandins.. Annu Rev Physiol.

[OCR_00643] Levine L., Hassid A. (1977). Epidermal growth factor stimulates prostaglandin biosynthesis by canine kidney (MDCK) cells.. Biochem Biophys Res Commun.

[OCR_00665] MORSON B. C. (1955). Carcinoma arising from areas of intestinal metaplasia in the gastric mucosa.. Br J Cancer.

[OCR_00652] Matsuda Y., Ogawa M., Sakamoto K., Yamashita S., Kanda A., Kohno M., Yoshida N., Nishijima J., Murata A., Mori T. (1991). Development of a radioimmunoassay for human group-II phospholipase A2 and demonstration of postoperative elevation.. Enzyme.

[OCR_00659] Matsukura N., Kawachi T., Sugimura T., Nakadate M., Hirota T. (1979). Induction of intestinal metaplasia and carcinoma in the glandular stomach of rats by N-alkyl-N'-nitro-N-nitrosoguanidines.. Gan.

[OCR_00669] Murata K., Egami H., Shibata Y., Sakamoto K., Misumi A., Ogawa M. (1992). Expression of blood group-related antigens, ABH, Lewis(a), Lewis(b), Lewis(x), Lewis(y), CA19-9, and CSLEX1 in early cancer, intestinal metaplasia, and uninvolved mucosa of the stomach.. Am J Clin Pathol.

[OCR_00676] Nakaguchi K., Nishijima J., Ogawa M., Mori T., Tojo H., Yamano T., Okamoto M. (1986). Purification and some properties of membrane-associated phospholipase A2 of human spleen.. Enzyme.

[OCR_00682] Nakano T., Ohara O., Teraoka H., Arita H. (1990). Glucocorticoids suppress group II phospholipase A2 production by blocking mRNA synthesis and post-transcriptional expression.. J Biol Chem.

[OCR_00688] Nakano T., Ohara O., Teraoka H., Arita H. (1990). Group II phospholipase A2 mRNA synthesis is stimulated by two distinct mechanisms in rat vascular smooth muscle cells.. FEBS Lett.

[OCR_00694] Nolan R. D., Danilowicz R. M., Eling T. E. (1988). Role of arachidonic acid metabolism in the mitogenic response of BALB/c 3T3 fibroblasts to epidermal growth factor.. Mol Pharmacol.

[OCR_00700] Okamoto M., Ono T., Tojo H., Yamano T. (1985). Immunochemical relatedness between secretory phospholipase A2 and intracellular phospholipase A2.. Biochem Biophys Res Commun.

[OCR_00706] Poulsen S. S., Nexø E., Olsen P. S., Hess J., Kirkegaard P. (1986). Immunohistochemical localization of epidermal growth factor in rat and man.. Histochemistry.

[OCR_00717] Robert A., Nezamis J. E., Lancaster C., Hanchar A. J. (1979). Cytoprotection by prostaglandins in rats. Prevention of gastric necrosis produced by alcohol, HCl, NaOH, hypertonic NaCl, and thermal injury.. Gastroenterology.

[OCR_00711] Robert A., Schultz J. R., Nezamis J. E., Lancaster C. (1976). Gastric antisecretory and antiulcer properties of PGE2, 15-methyl PGE2, and 16, 16-dimethyl PGE2. Intravenous, oral and intrajejunal administration.. Gastroenterology.

[OCR_00723] Satoh Y. (1988). Effect of live and heat-killed bacteria on the secretory activity of Paneth cells in germ-free mice.. Cell Tissue Res.

[OCR_00728] Segura D. I., Montero C. (1983). Histochemical characterization of different types of intestinal metaplasia in gastric mucosa.. Cancer.

[OCR_00733] Senegas-Balas F., Balas D., Verger R., de Caro A., Figarella C., Ferrato F., Lechene P., Bertrand C., Ribet A. (1984). Immunohistochemical localization of intestinal phospholipase A2 in rat paneth cells.. Histochemistry.

[OCR_00740] Shimada S., Maeno M., Misumi A., Takano S., Akagi M. (1987). Antigen reversion of glycogen phosphorylase isoenzyme in carcinoma and proliferative zone of intestinal metaplasia of the human stomach. An immunohistochemical study.. Gastroenterology.

[OCR_00531] Shimada T., Nakamura S. (1987). Cytochrome P-450-mediated activation of procarcinogens and promutagens to DNA-damaging products by measuring expression of umu gene in Salmonella typhimurium TA1535/pSK1002.. Biochem Pharmacol.

[OCR_00750] Tasumi H., Tojo H., Senda T., Ono T., Fujita H., Okamoto M. (1990). Immunocytochemical studies on the localization of pancreatic-type phospholipase A2 in rat stomach and pancreas, with special reference to the stomach cells.. Histochemistry.

[OCR_00762] Tojo H., Ono T., Kuramitsu S., Kagamiyama H., Okamoto M. (1988). A phospholipase A2 in the supernatant fraction of rat spleen. Its similarity to rat pancreatic phospholipase A2.. J Biol Chem.

[OCR_00766] Vadas P., Pruzanski W. (1986). Role of secretory phospholipases A2 in the pathobiology of disease.. Lab Invest.

[OCR_00775] Wilson D. E., Phillips C., Levine R. A. (1971). Inhibition of gastric secretion in man by prostaglandin A.. Gastroenterology.

[OCR_00780] Wright N. A., Pike C., Elia G. (1990). Induction of a novel epidermal growth factor-secreting cell lineage by mucosal ulceration in human gastrointestinal stem cells.. Nature.

[OCR_00771] van den Bosch H. (1980). Intracellular phospholipases A.. Biochim Biophys Acta.

